# Diagnostic and Management Challenges in Atypical Central Serous Chorioretinopathy Mimicking Vogt-Koyanagi-Harada Disease: A Case Report

**DOI:** 10.7759/cureus.91080

**Published:** 2025-08-26

**Authors:** Woan Shian See, Rajasudha Sawri Rajan, Sheena Mary Alexander

**Affiliations:** 1 Department of Ophthalmology, Hospital Selayang, Kuala Lumpur, MYS; 2 Department of Ophthalmology, Queen Elizabeth Hospital, Kota Kinabalu, MYS

**Keywords:** atypical central serous chorioretinopathy, exudative retinal detachment, steroid, vkh disease, vogt-koyanagi-harada

## Abstract

Central serous chorioretinopathy (CSCR) is characterized by serous detachment of the neurosensory retina and is considered part of the pachychoroid spectrum. Vogt-Koyanagi-Harada (VKH) disease, in contrast, is characterized by bilateral granulomatous panuveitis, exudative retinal detachments, and choroidal thickening. As both are primarily diseases of the choroid, the rare clinical overlap between atypical CSCR and VKH disease can complicate diagnosis. We report a case of atypical CSCR mimicking VKH disease, highlighting the diagnostic and treatment challenges.

A 50-year-old woman presented with bilateral progressive reduction in vision over three months, accompanied by headaches and tinnitus. Ocular examination revealed bilateral exudative retinal detachment, and she was diagnosed with acute incomplete VKH disease. Despite systemic corticosteroid therapy, her condition worsened, leading to a revised diagnosis of VKH disease with steroid-induced CSCR. Systemic corticosteroids were tapered and discontinued due to CSCR development. Subsequent surgical interventions, including vitreous biopsy, vitrectomy, subretinal fluid drainage, and silicone oil tamponade, were carried out. One month postoperatively, recurrent exudative retinal detachment occurred. With a suspicion of masquerade syndrome, she underwent left eye removal of oil, retinal and choroidal tissue biopsy, subretinal fluid sampling and drainage, endolaser, and silicone oil tamponade. Histopathological examination and biopsies revealed no malignancy, infection, or inflammation. Postoperatively, visual improvement was limited due to chronic exudative retinal detachment. Ultimately, the diagnosis was revised to atypical CSCR based on clinical findings, disease progression, and multimodal imaging.

Atypical bullous retinal detachment is a rare manifestation, emphasizing the need to consider this variant in cases of serous retinal detachment. Timely recognition is essential to guide appropriate treatment and minimize visual complications.

## Introduction

Central serous chorioretinopathy (CSCR) is an idiopathic chorioretinal disease that causes serous neurosensory and retinal pigment epithelium detachment (PED) [1,2]. A rare form of this condition, known as atypical bullous CSCR, was first described by Gass in 1973 [3]. It is characterized by an extensive bullous exudative retinal detachment.

Atypical bullous CSCR can often be misdiagnosed as conditions such as Vogt-Koyanagi-Harada (VKH), posterior scleritis, posterior uveitis, and masquerade syndrome [4]. This is due to its similar clinical finding of multifocal serous retinal detachment. However, failure to distinguish atypical bullous CSCR may worsen the disease, particularly with the inappropriate use of systemic corticosteroids and other immunosuppressive therapies.

In the present case, we report a patient with atypical bullous CSCR that was initially misdiagnosed as VKH, emphasizing the challenges in diagnosis and management.

## Case presentation

**Figure 1:**
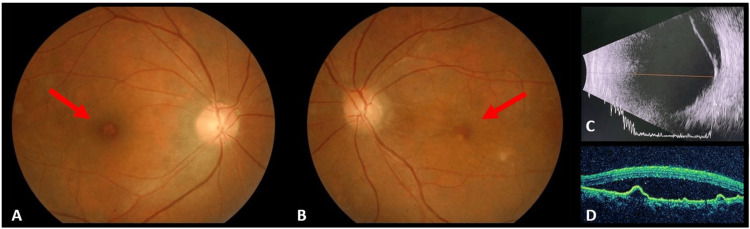
Fundus photography upon presentation. A) PED over the macula in the right eye. B) Serous retinal detachment over the macula with PED in the left eye. C) Retinal detachment in the inferior quadrants on USG of the left eye. D) Macular OCT section of the left eye showing subretinal fluid and multiple PEDs. PED: Pigment epithelial detachment; OCT: Optical coherence tomography.

A 50-year-old woman with a known history of hypertension, dyslipidemia, fatty liver disease, and anxiety disorder presented with progressive bilateral reduction in vision for 3 months. This was associated with headaches and tinnitus. On examination, her visual acuity was 6/10 in the right eye, while that in the left eye was reduced to 6/120. Anterior segment examination of both eyes was unremarkable, with intraocular pressure measuring 12 mmHg bilaterally. Funduscopic examination revealed exudative retinal detachment along the inferotemporal arcade in the right eye with a small PED overlying (Figure 1A). In the left eye, there was exudative retinal detachment over the inferior periphery and serous retinal detachment involving the posterior pole (Figure 1B). Both eyes showed no vitritis.

USG of the left eye demonstrated inferior retinal detachment (Figure 1C). There was no scleral thickening or T sign. Optical coherence tomography (OCT) of the right eye showed a PED over the fovea with subretinal fluid in the inferonasal quadrant, while the left eye demonstrated subretinal fluid tracking inferiorly with multiple PEDs and no subretinal septations (Figure 1D). Measurement of choroidal thickness was not performed due to the unavailability of an Enhanced Depth Imaging Optical Coherence Tomography (EDI-OCT) machine.

**Figure 2:**
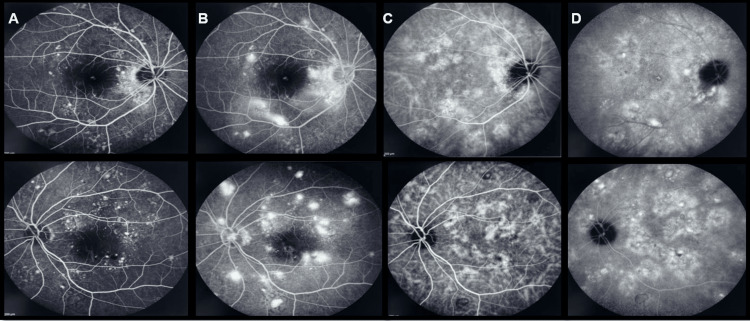
Multimodal imaging upon presentation. A) Fundus fluorescein angiography (FA) of both eyes revealed multiple pinpoint areas of early hyperfluorescence. B) FA of both eyes showed late staining in the macula and in the superotemporal and inferotemporal arcades. C) Indocyanine green angiography (ICGA) of both eyes revealed early hyperfluorescence with dilated choroidal vessels at the temporal vascular arcade. D) ICGA of both eyes showed increased hyperfluorescence in the macula and in the superotemporal and inferotemporal vascular arcades during the late phases.

Fundus fluorescein angiography (FA) revealed multiple pinpoint leakages without optic disc leakage, resembling the “starry sky appearance” seen in VKH (Figures 2A-2B). Indocyanine green angiography (ICGA) showed delayed choroidal filling and dilated choriocapillaris (Figures 2C-2D). A comprehensive systemic work-up for infections, inflammatory markers, and tumor markers was negative.

A preliminary diagnosis of incomplete VKH disease was made due to the presence of serous retinal detachment in both eyes, accompanying systemic symptoms such as tinnitus and headache, the presence of subretinal fluid on OCT, and multiple pinpoint leakages demonstrated on FA. The patient subsequently underwent a three-day course of IV pulse corticosteroid therapy, followed by oral prednisolone at a dose of 60 mg daily.

One month after treatment, her right visual acuity decreased to 6/20, and left visual acuity deteriorated to hand movement. Exudative retinal detachment progressed in both eyes. The patient was managed as a case of bilateral VKH with steroid-exacerbated CSCR, considered a complication of VKH treatment. The treatment goal was to control the underlying VKH with a second-line immunosuppressant while tapering systemic corticosteroids. Oral methotrexate 15 mg once weekly was prescribed, and oral steroids were rapidly tapered within one month.

**Figure 3:**
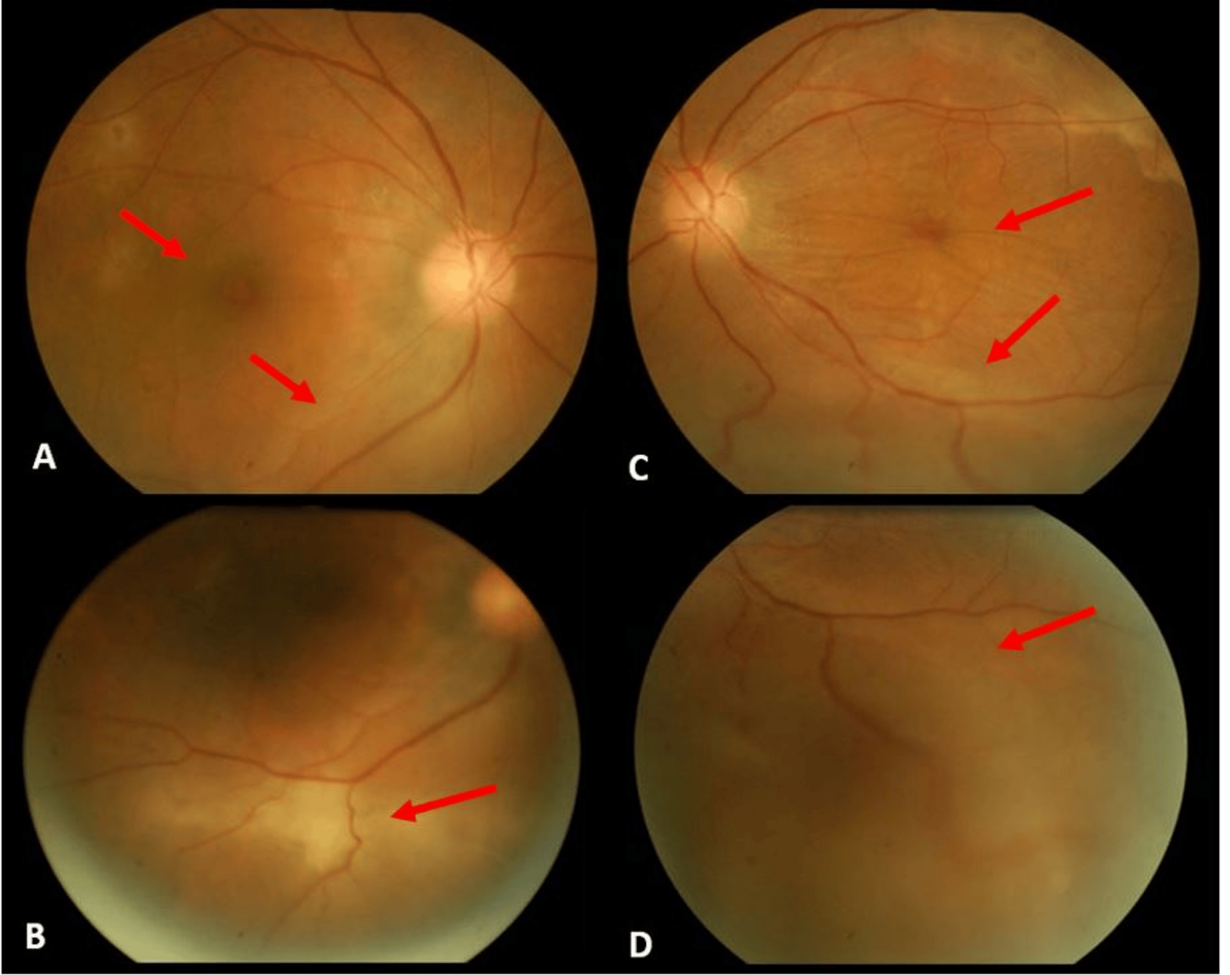
Fundus photography of both eyes after two months of systemic steroid and immunosuppressant therapy. A) Serous retinal detachment with subretinal deposits in the right eye. B) Subretinal fibrosis over the inferotemporal arcade in the right eye. C) Worsening serous retinal detachment over the macula and subretinal fibrosis over the superotemporal arcade in the left eye. D) Worsening bullous retinal detachment over the inferotemporal arcade in the left eye.

After two months of treatment, her visual acuity had not improved, and exudative retinal detachment progressed further (Figure 3). The patient was referred to the vitreoretinal team for intervention. She underwent left eye phacoemulsification with intraocular lens implantation, vitreous biopsy, pars plana vitrectomy (PPV), subretinal fluid drainage, endolaser, and silicone oil tamponade. Intraoperatively, massive bullous exudative retinal detachment with fibrovascular bands and subretinal deposits was observed.

Unfortunately, one month postoperatively, the left visual acuity remained unchanged. The vitreous biopsy yielded no results, and the patient developed recurrent retinal detachment. Given the unusually aggressive course and poor response to treatment, masquerade syndrome was suspected. The patient underwent left eye removal of the silicone oil, retinal and choroidal tissue biopsy, subretinal fluid sampling and drainage, endolaser, and reinsertion of silicone oil tamponade. Histopathological examination of the retinal and choroidal tissue biopsy showed no malignant changes, and vitreous biopsy and subretinal fluid sampling were negative for malignancy, infection, and inflammation. As there were no further signs of inflammation, methotrexate was gradually weaned off.

**Figure 4:**
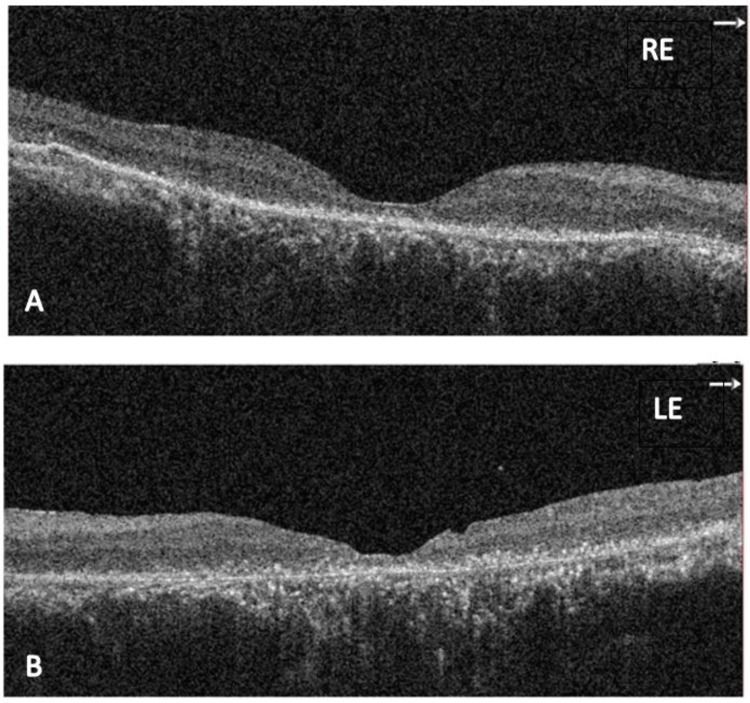
Optical coherence tomography (OCT) of both eyes after cessation of immunosuppressant therapy and post–pars plana vitrectomy of the left eye. (A-B) Foveal and retinal atrophy with an epiretinal membrane and thickened pachychoroid.

**Figure 5:**
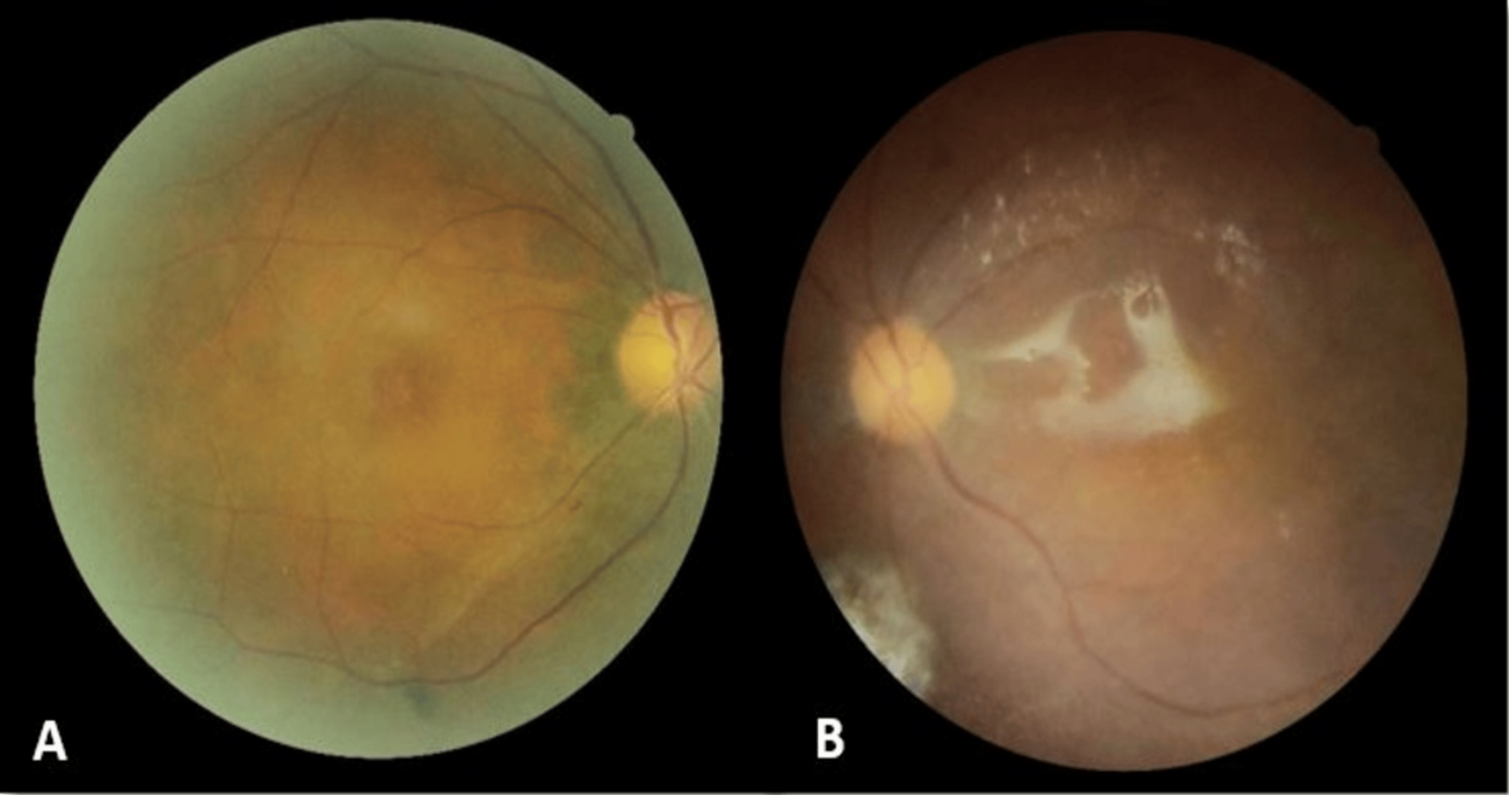
Fundus photography of both eyes after cessation of immunosuppressant therapy and post-pars plana vitrectomy of the left eye. A) Right eye showing an epiretinal membrane with resolved retinal detachment. B) Silicone oil-filled left eye with resolved retinal detachment and an epiretinal membrane.

Four months after cessation of treatment, the patient’s best corrected visual acuity (BCVA) was 6/120 in the right eye and hand movement in the left eye. Exudative retinal detachment had completely regressed in both eyes. However, there was an epiretinal membrane over the right eye and a macular scar with subretinal fibrotic bands in the left eye as sequelae of previous detachment. OCT of both eyes revealed retinal atrophy with a thickened pachychoroid (Figure 4). Based on the presentation, clinical findings, disease progression, and reinterpretation of previous multimodal imaging, the diagnosis was eventually revised to atypical bullous CSCR. One year later, the patient’s condition remained quiescent, with no reactivation of serous retinal detachments or signs of ocular inflammation. Fundus examinations revealed an epiretinal membrane in the right eye and a silicone oil-filled left eye with a macular scar and flat retina (Figure 5). No sunset glow fundus was observed.

## Discussion

Atypical bullous CSCR is a rare clinical variant of CSCR characterized by bilateral exudative retinal detachment and multiple foci of leakage. Risk factors for atypical CSCR include systemic steroid therapy, organ transplantation, haemodialysis, and pregnancy [3-5]. The pathophysiology of atypical CSCR remains unclear; however, choroidal vascular dysfunction plays a crucial role [3]. Studies have found that choroidal hyperpermeability alters and damages the RPE, leading to the formation of PED and subretinal exudation [3,5]. Consequently, subretinal exudation may result in exudative retinal detachment and the formation of a subretinal fibrotic membrane [3,5].

Bullous exudative retinal detachment can arise from various diseases such as VKH, posterior scleritis, posterior uveitis, and masquerade syndrome [3]. It is important to distinguish atypical bullous CSCR from these entities to avoid inappropriate initiation of systemic steroids.

Clinical findings that support the diagnosis of atypical CSCR include the absence of cells in the anterior chamber and vitreous, and the absence of systemic manifestations. Conversely, VKH typically presents with signs of intraocular inflammation such as anterior chamber cells, vitritis, and optic disc swelling. VKH is also usually accompanied by systemic symptoms such as headache, tinnitus, meningism, or integumentary changes like vitiligo or poliosis, which are absent in CSCR. Multimodal imaging findings further support the diagnosis: OCT in CSCR typically shows subretinal precipitates, PED, and a thickened pachychoroid. FA demonstrates multiple pinpoint leakages in the pre-arterial phase with late staining and absence of optic disc leakage, while Indocyanine Green Angiography (ICGA) may reveal dilated choroidal vessels and delayed filling of the choroidal circulation [3,6]. In contrast, OCT in VKH shows subretinal fluid, intraretinal fluid septation, bacillary detachments, and RPE undulations. FA in VKH reveals multiple pinpoint leaks in the arterial phase with optic disc leakage, while ICGA shows dark hypofluorescent spots corresponding to choroidal inflammation.

The initial step in treatment involves elimination of risk factors and discontinuation of any form of steroid therapy [6]. In chronic CSCR with focal leakage or diffuse choroidal hyperpermeability without large bullous detachments, photodynamic therapy (PDT) with verteporfin (half-dose or half-fluence protocols) serves as an effective first-line treatment to induce choroidal remodelling and resolution of subretinal fluid [3,7]. Focal laser or micropulse laser can be considered when a single extrafoveal leak is identified on angiography, but these modalities are unsuitable for multifocal leaks or extensive bullous detachments [3,7].

Surgical options, including transscleral drainage and internal drainage, can be offered in cases of extensive bullous retinal detachment not responding to conventional therapy and in circumstances where immediate anatomical reattachment is required [3,7,8]. Surgical drainage allows rapid reattachment, but it carries risks such as proliferative vitreoretinopathy (PVR), RPE tears, recurrent detachment, and limited visual prognosis when detachment and subretinal fibrosis are chronic. Ozdemir et al. reported a case of atypical bullous CSCR treated surgically with external drainage; however, due to recurrence of retinal detachment, PPV with internal drainage through a small retinotomy and endolaser was performed, resulting in improved visual acuity [7]. Kang et al. employed a similar surgical technique, and postoperatively the retina remained attached with improved visual acuity [9].

In this case, the initial diagnosis of bilateral VKH was supported by serous retinal detachment, systemic symptoms, subretinal fluid on OCT, and FA findings showing multiple pinpoint leakages, leading to corticosteroid therapy that exacerbated the condition. Lack of improvement and concern for masquerade syndrome prompted pars plana vitrectomy (PPV) for diagnostic purposes. Following PPV, the retina remained attached and the disease has remained stable to date. However, final best-corrected visual acuity (BCVA) did not improve significantly due to chronic detachment leading to irreversible RPE and photoreceptor atrophy. PDT was not attempted initially because of diagnostic uncertainty, as it is generally reserved for confirmed chronic CSCR with persistent leakage. The diagnosis of atypical CSCR was made retrospectively after review of the clinical course and imaging.

While PDT is supported by high-level evidence for chronic CSCR, management in this case aligned with lower-level evidence due to diagnostic ambiguity. Local resource constraints and the urgency to exclude sight-threatening causes also influenced treatment choices. Interestingly, anatomical resolution occurred after PPV, though its therapeutic role in CSCR remains speculative.

## Conclusions

In conclusion, this rare variant of CSCR posed significant diagnostic and treatment challenges. This case illustrates the importance of maintaining diagnostic flexibility. Early use of multimodal imaging, careful risk factor assessment, and multidisciplinary consultation may help prevent misdiagnosis. When the clinical course diverges from expectations, timely reassessment and revision of diagnosis are critical to avoid unnecessary or harmful interventions.

This case report serves as a reminder that clinicians should maintain a high index of suspicion for atypical bullous CSCR in cases of chronic exudative retinal detachment that do not fulfill the criteria of other pathologies. Early recognition of this condition is paramount to avoid inappropriate use of steroids, prevent disease progression, and mitigate visual complications.
